# β‑Caryophyllene-Loaded Sunflower Oil Nanoemulsion Incorporated into Chitosan Films for Wound Healing Applications

**DOI:** 10.1021/acsomega.6c02735

**Published:** 2026-06-23

**Authors:** Renata De Carvalho Feitosa, Beatriz Ketlyn Da Cunha Batista, Thais Mariana Bezerra Tavares, Thayse Emanuelle Félix Dos Santos, Addison Ribeiro De Almeida, Leandro De Santis Ferreira, Weslley De Souza Paiva, Hugo Alexandre De Oliveira Rocha, Anne Emmanuelle Camara Da Silva Melo, Matheus De Freitas Fernandes-Pedrosa, Arnóbio Antônio Da Silva Júnior

**Affiliations:** † Laboratory of Pharmaceutical Technology and Biotechnology (TecBioFar), Graduate Program in Pharmaceutical Sciences, Department of Pharmacy, Federal University of Rio Grande do Norte (UFRN), Natal, Rio Grande do Norte 59012-570, Brazil; ‡ Laboratory of Biotechnology of Natural Polymers (BIOPOL), Department of Biochemistry, Federal University of Rio Grande do Norte (UFRN), Natal, Rio Grande do Norte 59078-970, Brazil

## Abstract

Skin wounds, particularly chronic lesions, represent a major global clinical challenge, as current treatments remain limited in their ability to provide effective, accessible, and biologically active therapies that accelerate tissue repair. This challenge has driven increasing interest in the development of advanced wound dressings based on multifunctional biomaterials. In this study, we hypothesized possible combination of three natural compounds (two nonpolar and one hydrogel), with well-recognized healing properties, in a single multifunctional device. Thus, sunflower oil nanoemulsion containing a bicyclic sesquiterpene β-caryophyllene (BCP) was incorporated into chitosan-based dressing formulations as a potential wound-healing device. The optimized nanoemulsion containing 0.5% BCP presented a mean droplet diameter of about 114 nm, narrow size distribution (PdI = 0.136), and high encapsulation efficiency (96%). The presence of nanoemulsion induced formation of homogeneous and flexible chitosan films, with skin-compatible pH, and contained 13.3 mg BCP per g of multifunctional device. The dressing was characterized in terms of physicochemical, morphological, thermal, and mechanical properties, as well as in vitro biological activity. Spectroscopic and thermal analyses confirmed intermolecular interactions between the components and maintenance of thermal and structural stability. The BCP-loaded film displayed excellent cytocompatibility, antioxidant activity, and a pronounced stimulatory effect on L929 fibroblast migration, resulting in complete in vitro wound closure within 24 h. Overall, the device exhibits suitable structural integrity and in vitro biological performance, supporting its potential as a promising platform for topical wound-healing applications.

## Graphical abstract

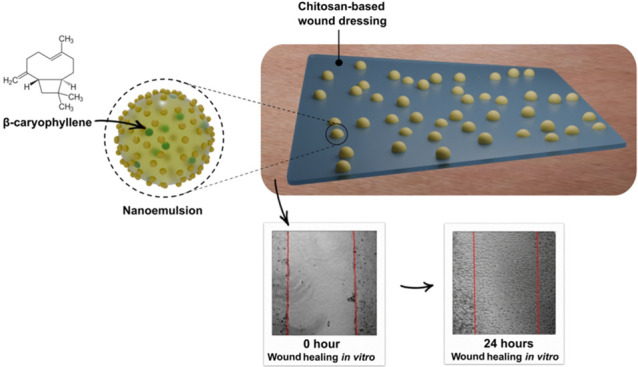

## 1 Introduction

Wound healing is a dynamic and multifactorial process involving the stages of hemostasis, inflammation, proliferation, and tissue remodeling. Dysregulation of these responses, particularly in inflammatory and oxidative mechanisms, can impair tissue regeneration, leading to chronic wounds that are difficult to treat.^1,2^ Therefore, there is a growing demand for therapeutic systems capable of modulating inflammation and promoting skin regeneration in an efficient and controlled manner.^3^ Natural polymer–based bioactive dressings have emerged as promising alternatives to conventional wound dressings, as they provide a favorable environment for tissue regeneration. Among these materials, chitosan has been extensively investigated due to its biocompatibility, biodegradability, antimicrobial and hemostatic properties.^4,5^ Moreover, its functional structure allows the incorporation of bioactive compounds, enhancing the therapeutic performance of the dressing.^6^

Sunflower oil and β-caryophyllene (BCP) are natural compounds with well-recognized wound-healing potential. The former contains linoleic acid, which restores lipid bilayer organization and ceramide synthesis via PPAR-α activation and further promotes angiogenesis and epithelialization during wound healing,^7^ while BCP, a bicyclic sesquiterpene acting as a selective agonist of cannabinoid type-2 receptors (CB2-R), exhibits anti-inflammatory activity and stimulates cell proliferation without psychotropic effects.^8^ However, the lipophilic nature of these compounds poses challenges for their incorporation and stability within hydrophilic polymeric matrices.

In this context, the use of nanoemulsions (NEs) represents an effective strategy for encapsulating and dispersing hydrophobic compounds in aqueous media, improving their stability and enhancing skin penetration.^9^ The combination of NEs with biopolymers such as chitosan has shown promising outcomes in tissue regeneration and microbial control.^10,11^ Therefore, this study proposes the development of an innovative bioactive dressing composed of a chitosan film containing a BCP-loaded sunflower oil-based NE, aiming to improve wound healing through a sustainable, biocompatible, and multifunctional advanced formulation.

## 2 Materials and Methods

### 2.1 Materials

All other reagents used in this study were purchased commercially and have adequate analytical grade. Low molecular weight chitosan (Mw 50,000–190 000 Da, viscosity 20–300 cps, degree of deacetylation 85%), β-Caryophyllene (purity ≥ 80%), polysorbate 80 (Tween 80), sorbitan monooleate (Span 80) were obtained from Sigma-Aldrich Co. (Saint-Louis, MO, USA). Sunflower oil was purchased from Ferquima (Brazil) and double distilled glycerin from Synth (Brazil). Purified water was obtained by reverse osmosis equipment, model OS50 LX (Gehaka, Brazil). L929 cells (ATCC CCL-1) were purchased from the America6n Type Culture Collection (ATCC) (Rockville, MD, USA).

### 2.2 Development of Nanoemulsions

The empty sunflower oil nanoemulsion (Blank-NE) was prepared using the phase inversion method by gradually dripping the aqueous phase into the oil phase under magnetic stirring (1500 rpm) at room temperature (25 °C), leading to phase inversion and formation of an oil-in-water (O/W) nanoemulsion system. After the dripping process was completed, the formulations were kept under continuous stirring for 30 min. The aqueous phase consisted of purified water, while the oil phase was composed of sunflower oil, glycerol (10% w/w), polysorbate 80, and sorbitan monooleate. The concentration of the oil and surfactant mixture varied from 0.5 to 2.5% (w/w), maintaining a 1:1 ratio (sunflower oil:surfactant mixture), with a fixed 75:25 ratio between polysorbate 80 and sorbitan monooleate. For film preparation, nanoemulsion batches were scaled up from 10 to 100 g, replacing magnetic stirring (1500 rpm) with mechanical blade stirring (1200 rpm) to ensure efficient mixing at higher volumes. The selection of the oil concentration for subsequent BCP loading was based on the maximum incorporation capacity observed after incorporation into chitosan films. β-caryophyllene-loaded NE (BCP-NE) were prepared using the same procedure, with the addition of BCP (0.5% w/w) to the oil phase. The final composition includes 1% (w/w) sunflower oil, 10% (w/w) glycerol, 1% (w/w) of the surfactant mixture (polysorbate 80:sorbitan monooleate, 75:25), and purified water q.s. to the final volume. For the preliminary screening and optimization of the NE composition, two independent preparations of each formulation were produced (*n* = 2). After selection of the optimized formulation, all subsequent experiments were conducted in triplicate.

### 2.3 Hydrodynamic Diameter and Zeta Potential

The hydrodynamic diameter and polydispersity index (PdI) of the developed formulations were evaluated by dynamic light scattering (DLS) using a Zetasizer Nano ZS analyzer (Malvern Instruments Ltd., UK). Samples were diluted with ultrapure water (1:100) and measured at 25 ± 2 °C, with a detection wavelength of 633 nm and a scattering angle of 173°. Zeta potential analysis was performed on the same instrument based on electrophoretic mobility, also at 25 ± 2 °C. The samples were analyzed 24 h, 7 days, 14 days, and 21 days after preparation. Results are expressed as mean ± standard deviation (*n* = 2).

### 2.4 Preparation of Chitosan Films (CH-Film, Blank-Film, and BCP-Film)

Control chitosan films (CH-Film), without nanoemulsions, were prepared using a 2% (w/v) chitosan solution dissolved in 60 mL of 2% (v/v) glacial acetic acid under magnetic stirring at 25 °C for 24 h to ensure complete polymer dissolution. The solution was then poured into glass Petri dishes (150 mm Ø) and dried in an oven at 40.0 ± 0.2 °C for 48 h to promote solvent evaporation (casting) and film formation. After drying, 100 mL of 1.0 M sodium hydroxide (NaOH) solution was added for 2 min. The films were then removed, rinsed with approximately 250 mL of distilled water to eliminate residual NaOH, and air-dried under controlled conditions (25 °C; 50% RH) for 24 h. Chitosan films containing unloaded sunflower oil-based nanoemulsions (Blank-Film) and β-caryophyllene-loaded nanoemulsions (BCP-Film) were prepared using a mechanical stirrer (RW 20 digital, IKA, Germany) equipped with a four-blade propeller (R 1342). The nanoemulsion oil phase was pre-homogenized at 600 rpm for 5 min, followed by dropwise addition of the aqueous phase (10 mL/min) under stirring at 1200 rpm and an additional 30 min of mixing. The stirring speed was then reduced to 400 rpm, and 2% (v/v) acetic acid and 2% (w/v) chitosan were added, followed by 10 min of stirring to ensure polymer dispersion. The formulations were subsequently subjected to magnetic stirring at 1,500 rpm for 24 h for complete chitosan dissolution and degassing. The resulting dispersions were cast into glass Petri dishes (50 g for 150 mm dishes and 10 g for 35 mm dishes) and dried at 30.0 ± 0.2 °C for 40 h. The films were then removed using a spatula, with no requirement for a release agent (Figure S1E).

### 2.5 Physicochemical, Mechanical, and Structural Characterization of Films

Film weight variation was determined using a precision balance (±0.001 g, Shimadzu), and thickness was measured using a digital micrometer (SIMOKIT). Circular samples (32 mm Ø) from each formulation were analyzed in sextuplicate, and the individual values were used to calculate mean weight, mean thickness, and standard deviation. Surface pH was measured using a Skin-pH-Meter (COURAGE+KAZAKA), with circular samples (32 mm Ø) evaluated in triplicate. Moisture content was determined using a semianalytical infrared moisture analyzer (i-THERMO 163-L, BEL, Italy), using approximately 1 g of each film per analysis, with automatic termination upon reaching constant weight. Wettability was assessed using a Krüss DSA100 goniometer with distilled water. A 5 μL droplet was deposited onto the film surface, and the contact angle was recorded using the instrument camera. Measurements were performed in duplicate for each formulation.

Fourier transform infrared (FTIR) spectroscopy was performed using an IR Prestige-21 spectrometer (Shimadzu) equipped with an attenuated total reflectance (ATR) accessory, in the mid-infrared region (700–4000 cm^–1^), at a resolution of 4 cm^–1^ with 20 scans. Scanning electron microscopy (SEM) analysis was carried out using a Carl Zeiss Auriga microscope at magnifications up to 8000× and an accelerating voltage of 5 kV. Prior to imaging, the films were sputter-coated with a thin conductive metal layer using a Sputter Coater (SCD 005, BAL-TEC). X-ray diffraction (XRD) patterns were obtained using an X’Pert-MPD diffractometer (Philips Analytical X-ray, Netherlands) equipped with Cu Kα radiation (λ = 1.54056 Å), operating at 40 kV and 40 mA over a 2θ range of 5–50°, with a step size of 0.050° and a scan rate of 0.033°/s. Thermogravimetric analysis (TGA) and differential scanning calorimetry (DSC) were performed using a thermal analyzer (TGA/DSC1, Mettler Toledo, Switzerland). Samples were heated from 25 to 300 °C at a rate of 10 °C/min under a nitrogen flow of 50 mL/min. Transmission electron microscopy (TEM) was used to evaluate the morphology of the nanoemulsion droplets prior to incorporation into the films. Samples were diluted (1:20, v/v) in purified water, deposited onto copper grids, and air-dried at room temperature before analysis using a FEI Tecnai G2 Spirit Biotwin microscope operating at 120 kV.

The mechanical properties of the Blank-Film and BCP-Film were evaluated using a TA.XT2i texture analyzer (Stable Micro Systems, Godalming, UK) equipped with a 5 mm ball-end puncture probe. The films were fixed between two plates with a central circular opening (10 mm diameter) using pins to ensure stability and centrally positioned for testing. Measurements were performed at a constant crosshead speed of 0.1 mm s^–1^ and a trigger force of 0.005 kg under ambient conditions. Force–displacement curves were recorded until film rupture. The maximum force at rupture was defined as puncture resistance (PR, N), and elongation at break (EB, %) was calculated according to eq 1:

$$\mathrm{EB}=\frac{\sqrt{r^{2}+d^{2}}-r}{r}\times 100$$ 1

where *r* is the probe radius (5 mm) and *d* is the displacement at rupture (mm).

### 2.6 GC–MS Calibration Curve for β-Caryophyllene

The calibration curve was constructed from the triplicate analysis of standard BCP solutions. A 2 mg amount of standard was weighed and used to prepare stock solutions at a concentration of 1 mg/mL. These were subsequently diluted with *n*-hexane to obtain working solutions ranging from 0.001 to 0.04 mg/mL. Analyses were carried out using a gas chromatograph model 5977B MSD (Agilent), equipped with an HP-5MS capillary column (30 m × 0.25 mm × 0.25 μm, Agilent). Analytical conditions were as follows: injection volume of 1 μL in splitless mode; electron ionization at 70 eV; helium as the carrier gas at a flow rate of 1 mL/min; injector temperature 250 °C; ion source temperature 230 °C; and transfer line temperature 280 °C. The total run time was 17.8 min, with data acquisition beginning at 5 min. The oven temperature program started at 110 °C, increased at 15 °C/min to 160 °C, then ramped at 20 °C/min to 290 °C, where it was held for 5 min. The acquisition mode was full scan in the *m*/*z* range of 50–750.

### 2.7 β-Caryophyllene Extraction and Analytical Preparation

To determine BCP content in the NEs, 1 mL of the formulation was transferred into a test tube and extracted with 5 mL of *n*-hexane. The extraction was performed using vortex agitation for 5 min, followed by 15 min in an ultrasonic bath. This cycle was repeated five times for each sample to ensure exhaustive recovery of BCP. After the final extraction, 20 μL of the hexane phase (supernatant) was transferred to a vial containing 980 μL of hexane for subsequent GC-MS analysis, as described in Section 2.8. For quantification in the films, circular samples (32 mm diameter, in triplicate) were weighed individually. Each film was placed into a 50 mL beaker and dissolved in 40 mL of 2% acetic acid solution under magnetic stirring for 24 h. Then, 1 g of the resulting solution was transferred to a test tube, and BCP was extracted using 5 mL of *n*-hexane following the same extraction procedure as for the NEs (vortex for 5 min, ultrasonic bath for 15 min, repeated 5 times). Subsequently, 50 μL of the supernatant was diluted with 950 μL of *n*-hexane in a vial and analyzed by GC-MS as described above. BCP concentrations were calculated using the external calibration curve described in Section 2.8.

### 2.8 Biological In Vitro Assays

The experimental groups were defined according to the purpose of each assay. For hemolysis and antioxidant assays, free BCP, Blank-NE, and BCP-NE were evaluated to assess the effects of BCP and the NE system, as well as any contribution from the formulation without the active compound. For cytocompatibility and in vitro wound healing assays, Blank-film and BCP-film were additionally included to assess the impact of incorporating the NE into the chitosan matrix and to compare the performance of the individual components with that of the final formulation.

#### 2.8.1 Total Antioxidant Capacity (TAC)

The total antioxidant capacity of the formulations was evaluated at two concentrations, 10 and 100 μg/mL, using the phosphomolybdenum method described by Prieto et al. (1999). Test tubes containing the samples and the reagent solution (0.6 M sulfuric acid, 28 mM sodium phosphate, and 4 mM ammonium molybdate) were incubated at 100 °C for 90 min. After cooling to room temperature, the absorbance of each solution was measured at 695 nm. Results were expressed as milligrams of ascorbic acid equivalent per gram of sample (mg AAE/g), and statistical analyses were performed using Welch’s ANOVA followed by the Games–Howell post hoc test.

#### 2.8.2 Hemolytic Assay

The in vitro hemolytic potential was evaluated using freshly collected human blood (type O^+^) from healthy donors, following the Declaration of Helsinki and approved by the Human Research Ethics Committee of the Onofre Lopes University Hospital, Federal University of Rio Grande do Norte (HUOL/UFRN), protocol no. 2.809.485. Blood in EDTA tubes (1.5 mg/mL) was centrifuged at 2,000 rpm for 10 min to separate erythrocytes, which were washed three times with 0.9% NaCl and resuspended (1:1, v/v). In microtubes containing 300 μL saline, 300 μL of BCP solution (5 mg/mL), Blank-NE, or BCP-NE (5 mg/mL) were added in six serial dilutions, followed by 20 μL of erythrocyte suspension. Distilled water and saline were used as positive and negative controls, respectively. Samples were incubated at 37 °C for 60 min, centrifuged at 2000 rpm for 10 min, and 200 μL of supernatant transferred to a 96-well plate for absorbance measurement at 540 nm (Epoch, BioTek Instruments, Winooski, VT). Hemolysis percentage was calculated based on hemoglobin release,^12^ and statistical analysis was performed by ANOVA followed by Tukey’s post hoc test.

The percentage of hemolysis was calculated according to eq 2:

$$\%\mathrm{Hemolysis}=\frac{\mathrm{ABSs}\;\mathrm{ample}-\mathrm{ABS}\;\mathrm{negative}\;\mathrm{control}}{\mathrm{ABS}\;\mathrm{positivecontrol}-\mathrm{ABS}\;\mathrm{negative}\;\mathrm{control}}\times 100$$ 2

where ABS = absorbance.

#### 2.8.3 Cytocompatibility Assay in L929 Fibroblasts

L929 fibroblasts (ATCC CCL-1) were seeded in 96-well plates (5 × 10^3^ cells/well) and cultured in DMEM with FBS for 24 h at 37 °C, 5% CO_2_ to allow adhesion. The medium was then replaced with serum-free DMEM for 24 h, followed by 100 μL DMEM with 10% FBS containing different sample concentrations: Blank-NE and BCP-NE (6.25–100 μg/mL), Blank-Film and BCP-Film (62.5–990 μg/mL, in 2% acetic acid), free BCP (0.03–0.5 μg/mL), and sunflower oil (0.06–1.0 μg/mL). Hydrophobic compounds were first solubilized in acetone (1000 μg/mL) and then diluted in water to prepare secondary stock solutions (6.3 μg/mL BCP; 10 μg/mL sunflower oil) for culture medium dilutions. After 24 h incubation, cell viability was assessed by MTT assay (1 mg/mL in DMEM). Following 4 h incubation, supernatants were replaced with 100 μL ethanol to solubilize formazan, agitated for 15 min at 25 °C, and absorbance measured at 570 nm. Results were expressed as percentage MTT reduction relative to negative control, and data as mean ± SD from three independent experiments in triplicate. Different concentration ranges were applied in MTT and hemolysis assays due to the distinct biological models and assay sensitivities.

#### 2.8.4 Wound Healing (Scratch) Assay

L929 cells (ATCC CCL-1) were seeded in 24-well plates and cultured in DMEM containing 10% FBS until reaching approximately 80–90% confluence. Cells were then incubated for an additional 24 h at 37 °C in a 5% CO_2_ atmosphere. A uniform scratch was created using a sterile 200 μL pipet tip, and detached cells were removed by washing with PBS (pH 7.4). The wounded monolayers were treated with Blank-NE (100 μg/mL), BCP-NE (100 μg/mL), Blank-Film (990 μg/mL), and BCP-Film (990 μg/mL), both previously diluted in 2% acetic acid, as well as Free BCP (0.5 μg/mL). At the selected concentrations, BCP-NE and BCP-Film contained 0.5 μg/mL of BCP in their compositions. Wells containing only culture medium served as negative controls. To monitor wound closure, images were captured at 0, 12, and 24 h after treatment using a Nikon Eclipse inverted microscope equipped with a 10× objective lens. The wound area was analyzed using NIS-Elements AR software, and results were expressed as the percentage of wound closure relative to the initial area.

## 3 Results

### 3.1 Development and Characterization of Nanoemulsions

The development of NE and film was conducted in an integrated manner to determine the maximum oil content compatible with the polymer matrix of chitosan film. Oil-in-water (O/W) NEs were prepared by the phase inversion method using polysorbate 80 and sorbitan monooleate (75:25) as surfactants and glycerol as a cosolvent. The effect of varying sunflower oil concentration (0.5–2.5%) was investigated while maintaining a fixed 1:1 oil-to-surfactant ratio. The physicochemical properties of the formulations are presented in Table 1. All formulations exhibited a homogeneous visual appearance with no signs of instability after 24h, showing an average droplet diameter below 200 nm, negative zeta potential, and pH values ranging from 5.0 to 5.5. Sample A showed a higher PdI and a bimodal droplet size distribution, whereas formulations B, C, and D exhibited more suitable characteristics for further incorporation into chitosan films.

**Table 1:** Physicochemical Properties of Blank-NEs at Different Oil Concentrations^a^

sample	sunflower oil (%)	diameter (nm)	PdI	zeta-potential (mV)	pH
A	0.5	190.9 ± 5.9	0.427 ± 0.024	–37.9 ± 2.6	5.52 ± 0.02
B	1.0	108.3 ± 3.8	0.177 ± 0.019	–10.7 ± 1.4	5.41 ± 0.04
C	2.5	153.1 ± 12.2	0.338 ± 0.028	–28.5 ± 1.9	5.48 ± 0.02
D	5.0	158.9 ± 14.7	0.230 ± 0.070	–20.0 ± 2.7	5.06 ± 0.03

a Notes: PDI: polydispersity index; ζ-potential: zeta potential. All analyses were performed 24 h after sample preparation. Data are expressed as mean ± standard deviation (*n* = 2).

The corresponding Blank-Films revealed that formulations containing 2.5 and 5.0% sunflower oil exhibited surface oil droplets, suggesting that the oil content exceeded the incorporation capacity of the polymer matrix (Figure S1C,D). In contrast, the film containing 1.0% oil showed complete incorporation without visible surface oil (Figure S1B), indicating better compatibility with the chitosan matrix. Based on these results, the Blank-NE containing 1.0% sunflower oil was selected for subsequent BCP incorporation, with 0.5% (w/w) BCP added to the oil phase to obtain the BCP-NE. The formulation was then produced at a larger batch size to enable film fabrication. The physicochemical properties of the scaled-up formulations were evaluated (Table 2), showing no significant changes compared to the initial batches, confirming the robustness of the system upon scale-up, consistent with its composition-dependent nature.

**Table 2:** Physicochemical Characteristics of the NE-Blank and NE-BCP Formulations^a^,^b^

sample	BCP (%)	diameter (nm)	PdI	ZP (mV)	pH	EE (%)
blank-NE		115.5 ± 10.7	0.117 ± 0.059	–25.5 ± 2.3	5.41 ± 0.05	
BCP-NE	0.5	114.0 ± 3.2	0.136 ± 0.013	–27.8 ± 0.4	5.71 ± 0.02	96.0 ± 6.2

a Samples prepared using a mechanical blade stirrer.

b Notes: PdI: polydispersity index; ZP: zeta potential. All analyses were performed 24 h after sample preparation. Data are expressed as mean ± standard deviation (*n* = 3).

The resulting NEs were liquid, homogeneous, and whitish (Figure 1A), showing a monomodal droplet size distribution (Figure 1B). Regarding the BCP dosing method, linear calibration curves were obtained within the evaluated concentration range (0.001 to 0.04 mg/mL) for the BCP standard, which exhibited a retention time of 7.3 min under this method. The linearity coefficient was *R* ^2^ = 0.9942, with the regression equation *y* = 551814*x* + 43623. The method proved selective for the tested compound, with no peak overlap or interference observed at the BCP retention time (Figure 1C). The BCP-NE showed an encapsulation efficiency of 96%.

**Figure 1:**
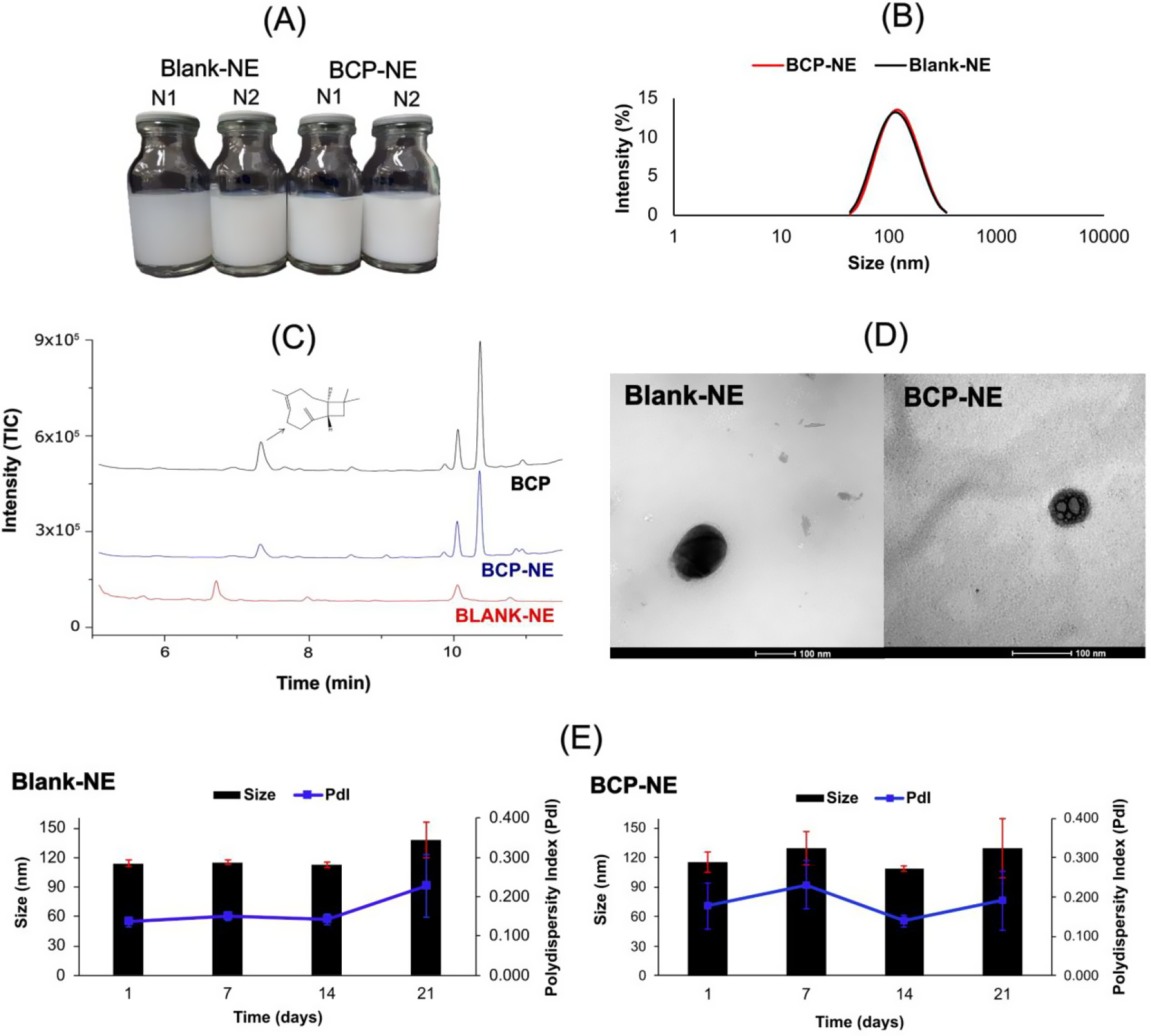
(A) Blank-NE and BCP-NE nanoemulsions, shown in duplicate. (B) Droplet size distribution based on intensity. (C) Gas chromatograms of free BCP, BCP-NE, and Blank-NE. (D) Transmission electron microscopy images. (E) Droplet size and polydispersity index over 21 days. Values are mean ± SD (*n* = 3).

Transmission electron microscopy images revealed spherical droplets at the nanoscale, confirming the formation of the nanoemulsions. The images highlighted morphological differences between formulations with and without BCP. The control nanoemulsion (Blank-NE) displayed spherical lipid droplets with well-defined contours and homogeneous electron density, indicating uniform dispersion of sunflower oil and excellent colloidal stability. In contrast, BCP-NE showed droplets with heterogeneities in the lipid matrix, suggesting that BCP was incorporated into the oil phase and possibly associated with the formation of small compartments of distinct electron density within the droplets (Figure 1D).

Monitoring of the mean droplet size and PdI of Blank-NE and BCP-NE over 21 days demonstrated maintenance of colloidal stability, with homogeneous distribution and diameters around 100 nm, showing no significant changes over time. The presence of BCP did not induce relevant changes in droplet size or uniformity. Moreover, the PdI remained below 0.3, confirming the adequate homogeneity of the population (Figure 1E).

### 3.2 Cytocompatibility and Antioxidant Activity of Nanoemulsions

The cytocompatibility of Blank-NE and BCP-NE was evaluated in L929 fibroblasts using the MTT reduction assay. BCP concentrations in the NEs of 6.25, 12.5, 25, 50, and 100 μg/mL corresponded to 0.03, 0.06, 0.13, 0.26, and 0.51 μg/mL of BCP, respectively. Cell viability for Blank-NE remained above 80% at all tested concentrations, with no statistically significant differences observed by Welch ANOVA followed by the Games-Howell post hoc test (*P* > 0.05). BCP-NE showed a concentration-dependent reduction in viability, ranging from approximately 99.0% at 6.25 μg/mL to 75.3% at 100 μg/mL, with significant differences detected only for 100 μg/mL relative to 6.25 μg/mL (*P* = 0.0184) and 12.5 μg/mL (*P* = 0.0212) (Figure 2A,B).

**Figure 2:**
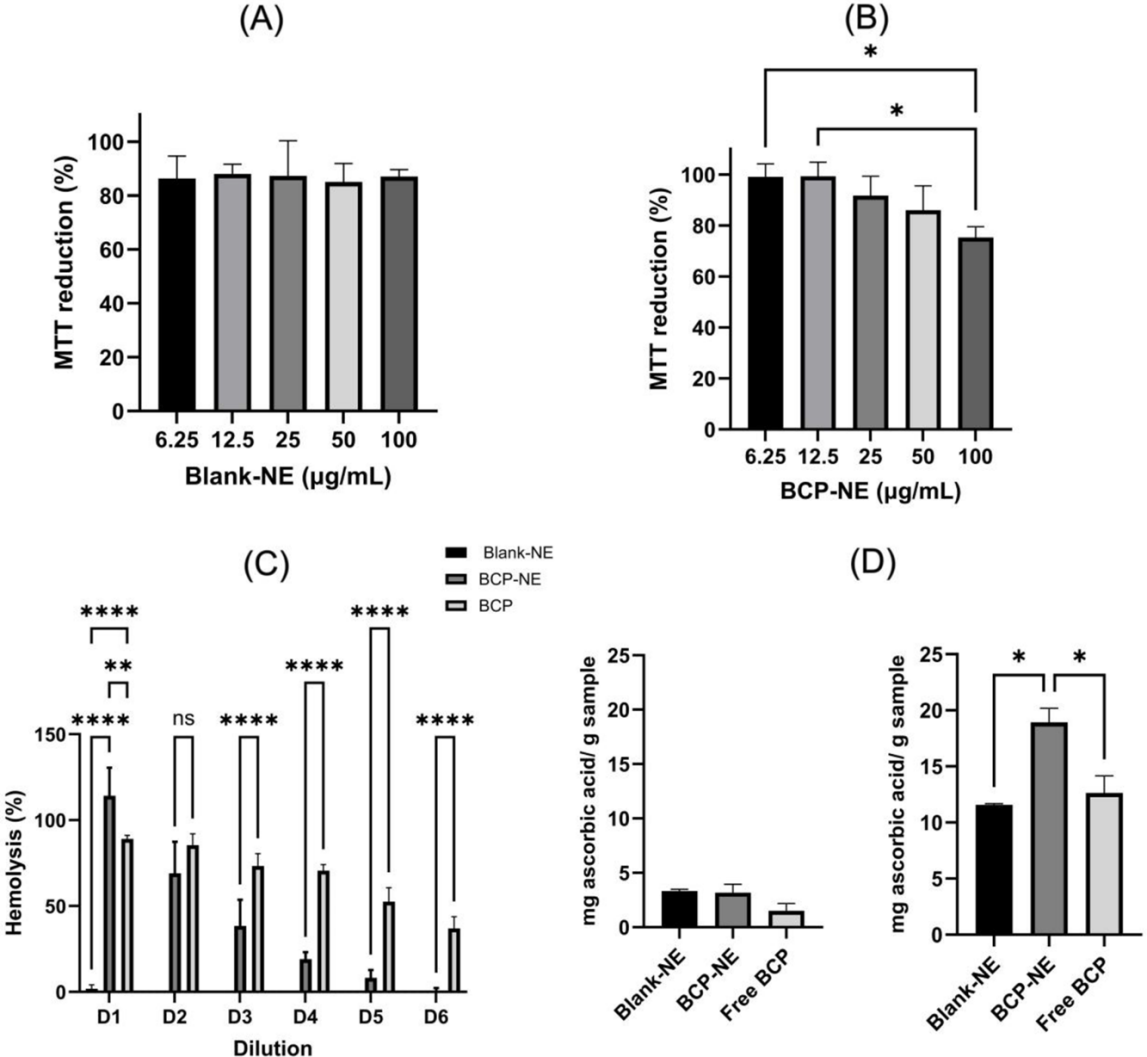
(A) Cytocompatibility of Blank-NE in L929 fibroblasts (MTT assay). (B) Cytocompatibility of BCP-NE in L929 fibroblasts (MTT assay). (C) Hemolytic profiles of the formulations. D1–D6 correspond to serial dilutions; for BCP-containing samples, these represent concentrations of 2.4, 1.2, 0.6, 0.3, 0.15, and 0.075 mg/mL of BCP, respectively, while Blank-NE was diluted proportionally. (D) Antioxidant activity of the formulations at 10 μg/mL (left) and 100 μg/mL (right). Data are expressed as mean ± SD (*n* = 3). *p < 0.05; **p < 0.01; ****p < 0.0001.

The in vitro hemolysis assay was performed to assess the safety of formulations potentially in contact with blood. Hemolytic profiles of Blank-NE, BCP-NE, and free BCP solution were evaluated across six dilutions corresponding to 2.4, 1.2, 0.6, 0.3, 0.15, and 0.075 mg/mL of BCP for BCP-containing samples. Free BCP exhibited high hemolysis at the highest concentration (89% at 2.4 mg/mL), decreasing to approximately 37% at the lowest (0.075 mg/mL). BCP-NE also induced 100% hemolysis at 2.4 mg/mL but dropped sharply at lower concentrations, reaching <10% at 0.15 mg/mL. In contrast, Blank-NE showed negligible hemolytic activity (<5%) at all concentrations, including 2.4 mg/mL, demonstrating excellent hemocompatibility of the base formulation (Figure 2C).

Total antioxidant activity of Blank-NE, BCP-NE, and free BCP was assessed at 10 and 100 μg/mL. At 10 μg/mL, no significant differences were observed between formulations: Blank-NE vs BCP-NE (*P* = 0.9387), Blank-NE vs BCP (*P* = 0.0658), and BCP-NE vs BCP (*P* = 0.1026). At 100 μg/mL, BCP-NE exhibited significantly higher antioxidant activity compared to Blank-NE (*P* = 0.0170) and BCP (*P* = 0.0125), while no significant difference was found between Blank-NE and BCP (*P* = 0.5594), suggesting a concentration-dependent effect on the observed antioxidant activity (Figure 2D).

### 3.3 Physicochemical, Mechanical, and Morphological Characterization of the Films

The films were prepared using the optimized NE formulation selected in the previous section. The chitosan films showed significant differences in their properties depending on their composition. The CH-Film exhibited lower mass (0.113 g) and thickness (0.128 mm) when compared to the Blank-Film (0.391 g; 0.505 mm) and the BCP-Film (0.464 g; 0.529 mm), showing that the addition of sunflower oil and BCP contributed to the increase in these structural characteristics. The pH remained slightly acidic (5.9–6.3), suitable for topical applications, while the moisture content remained similar between formulations (15.19–17.47%), suggesting that lipid incorporation did not significantly influence water retention. Quantitative analysis confirmed the effective incorporation of BCP, with a content of 13.3 ± 0.03 mg/g of film (Table 3; Figure S2).

**Table 3:** Physicochemical Characteristics of Films^a^

**sample**	**mass (g)**	**thickness (mm)**	**pH**	**moisture (%)**	**contact angle (°)**	**[BCP] (mg/g film)**
CH-Film	0.113 ± 0.010	0.128 ± 0.029	5.9 ± 0.2	16.56 ± 1.60	75.2 ± 0.35	
blank-Film	0.391 ± 0.027	0.505 ± 0.025	6.2 ± 0.1	17.47 ± 3.17	47.4 ± 9.69	
BCP-Film	0.464 ± 0.034	0.529 ± 0.031	6.3 ± 0.1	15.19 ± 2.09	46.0 ± 0.78	13.3 ± 0.03

a Notes: Mass and thickness measurements were performed using 32 mm Ø circular film specimens from each formulation.

In the wettability analysis, statistically significant differences were observed for the contact angles with water (*p* < 0.05). CH-Film presented a higher mean value (75.2°), indicating greater hydrophobicity, while Blank-Film (47.4°) and BCP-Film (46.0°) exhibited significantly lower angles, with no statistical difference between them, indicating greater affinity for water (Table 3; Figure 3–line II).

**Figure 3:**
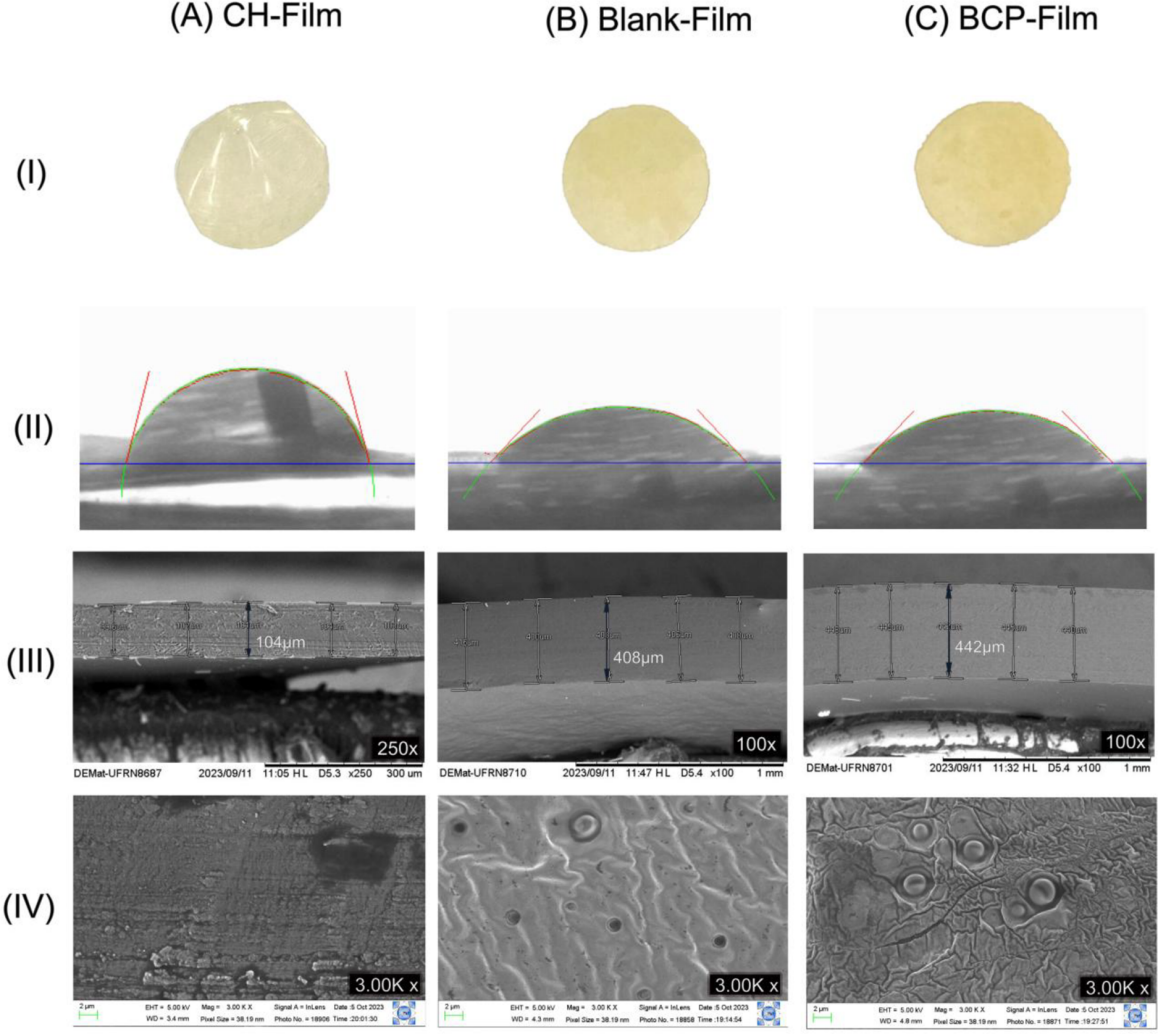
Morphological and surface characterization of CH-Film (column A), Blank-Film (column B), and BCP-Film (column C). Row (I): Circular 32 mm diameter film specimens on a white background. Row (II): Water contact angle measurements. Row (III): SEM images of film cross sections. Row (IV): Higher magnification cross-sectional images showing details of the internal structure.

The films exhibited a homogeneous appearance and circular shape, with a diameter of 32 mm, as illustrated in Figure 3 (row I). The CH-Film showed a translucent color, while the Blank-Film and BCP-Film displayed a yellowish hue, suggesting the incorporation of lipophilic components into the polymeric matrix. Cross-sectional images obtained by scanning electron microscopy (Figure 3–row III) confirmed that the CH-Film has a lower thickness compared to the films containing the lipid nanodispersion. At higher magnification (Figure 3–row IV), it was observed that the CH-Film presents a smooth and compact surface, whereas the Blank-Film and BCP-Film exhibit an internal morphology characterized by corrugated, rough, porous, and heterogeneous structures, with the presence of droplets dispersed within the matrix.

The mechanical properties of BCP-film and Blank-film were evaluated by puncture testing. The displacement at rupture was 7.01 ± 0.99 mm for BCP-film and 6.11 ± 1.70 mm for Blank-film, with higher variability observed for Blank-film. This is supported by the force–displacement curves, where one Blank-film replicate exhibited premature rupture (Figure S3). The force required for rupture was 137.80 ± 9.68 g (1.35 ± 0.09 N) for BCP-film and 135.76 ± 51.86 g (1.33 ± 0.51 N) for Blank-film. Puncture resistance (*R*_p_) values were 0.0795 ± 0.0034 MPa and 0.0969 ± 0.0125 MPa for BCP-film and Blank-film, respectively, with no statistically significant differences between formulations (*p* > 0.05). Elongation at break (EB) was 72.5 ± 16.4% for BCP-film and 59.0 ± 25.1% for Blank-film. Although no significant differences were observed, BCP-film showed lower variability, suggesting more uniform mechanical behavior.

The infrared (FTIR) spectra of Blank-Film and BCP-Film showed very similar profiles, reflecting the composition of the chitosan and sunflower oil matrix present in both formulations. Typical chitosan bands were identified, including Amide I (1650 cm^–1^) and Amide II (1550 cm^–1^), as well as C–O vibrations (1030 cm^–1^) attributed to the polysaccharide structure. Characteristic sunflower oil signals were also observed, such as the C=O stretching of esters (1745 cm^–1^) and methyl group bands (1460 cm^–1^). The spectrum of pure BCP exhibited bands associated with C=C double bonds (1630 cm^–1^) and out-of-plane C–H deformations (880 cm^–1^), typical of terpenes. However, these absorptions were not clearly visible in the BCP-Film spectrum, likely due to the dilution of the compound in the matrix, overlapping bands with vibrational groups from chitosan and sunflower oil, and the inherent limitations in depth and sensitivity of the technique (Figure 4A).

**Figure 4:**
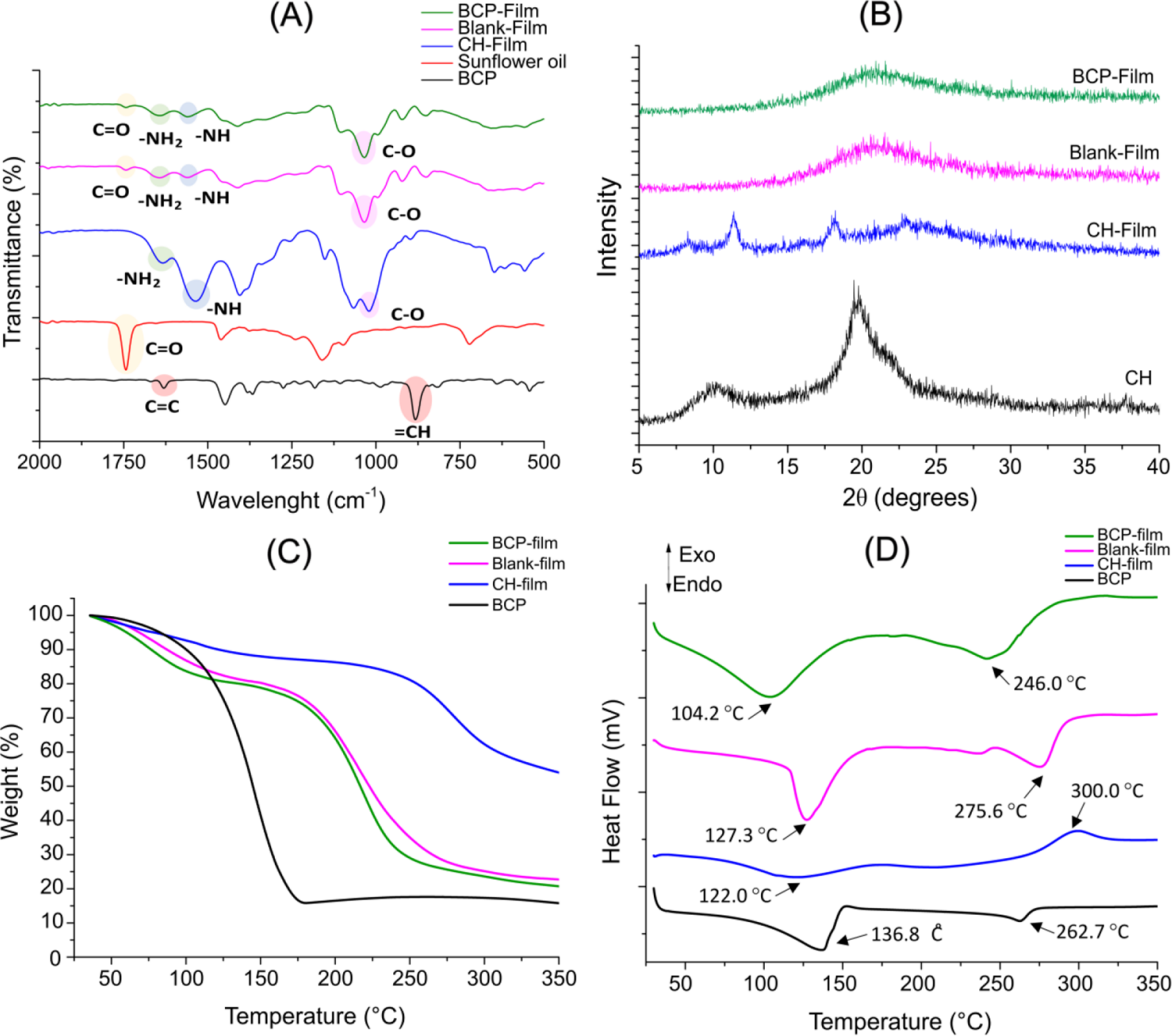
(A) FTIR spectra, (B) X-ray diffractograms, (C) thermograms (TGA), and (D) thermograms (DSC) of the films produced.

Despite these limitations, gas chromatography (GC) analysis confirmed the incorporation of BCP into the film (Figure S2), and morphological analysis by TEM (Figure 1D) suggested the incorporation of BCP into the oil phase, as evidenced by the presence of small compartments with distinct electron density within the droplets.

Additionally, X-ray diffraction (XRD) analysis showed that pure chitosan exhibits crystalline peaks at approximately 10° and 20° (2θ), consistent with its semicrystalline nature. The processed chitosan film displayed peaks shifted to 11° and 18° (2θ), whereas the Blank-Film and BCP-Film did not exhibit crystalline peaks, indicating that they are amorphous materials (Figure 4B).

Thermoanalytical analyses by TGA and DSC were performed to evaluate the thermal stability and transition events of the formulations. Free BCP showed rapid mass loss starting at approximately 100 °C, reaching about 80% degradation by 175 °C, which is typical behavior for volatile terpene compounds. In DSC, it exhibited an endothermic event between 125–150 °C (*T*_p_ = 136.8 °C) and another at 262.7 °C. CH-Film demonstrated high thermal stability up to 200 °C, with mass loss below 20%, while DSC revealed an endothermic peak at 122 °C and an exothermic peak at 300 °C. Blank-Film exhibited a gradual mass loss of 33.7% up to 200 °C and two endothermic events at 127.3 and 275.6 °C. Finally, BCP-Film showed a mass loss of 35.7% at 200 °C and two endothermic peaks at approximately 104.2 and 246 °C (Figure 4C,D).

### 3.4 Cytocompatibility and In Vitro Wound Healing Activity of the Films

The cytocompatibility of the formulations was evaluated using the MTT assay in L929 cells, with results expressed as the percentage of MTT reduction. Sunflower oil, Blank-NE, and Blank-Film samples preserved cell viability above 80% at all concentrations tested (Figure 5A). Statistical analysis revealed significant differences at a concentration of 0.13 μg/mL between sunflower oil and Blank-NE (*p* = 0.0231) and at 0.50 μg/mL, both between sunflower oil and Blank-NE (*p* = 0.0099) and between sunflower oil and Blank-Film (*p* = 0.0023). Sunflower oil showed cell viability greater than 100% at all tested concentrations, reaching a maximum of 144.7% at 0.50 μg/mL, while the Blank-NE and Blank-Film formulations showed values close to 100%.

**Figure 5:**
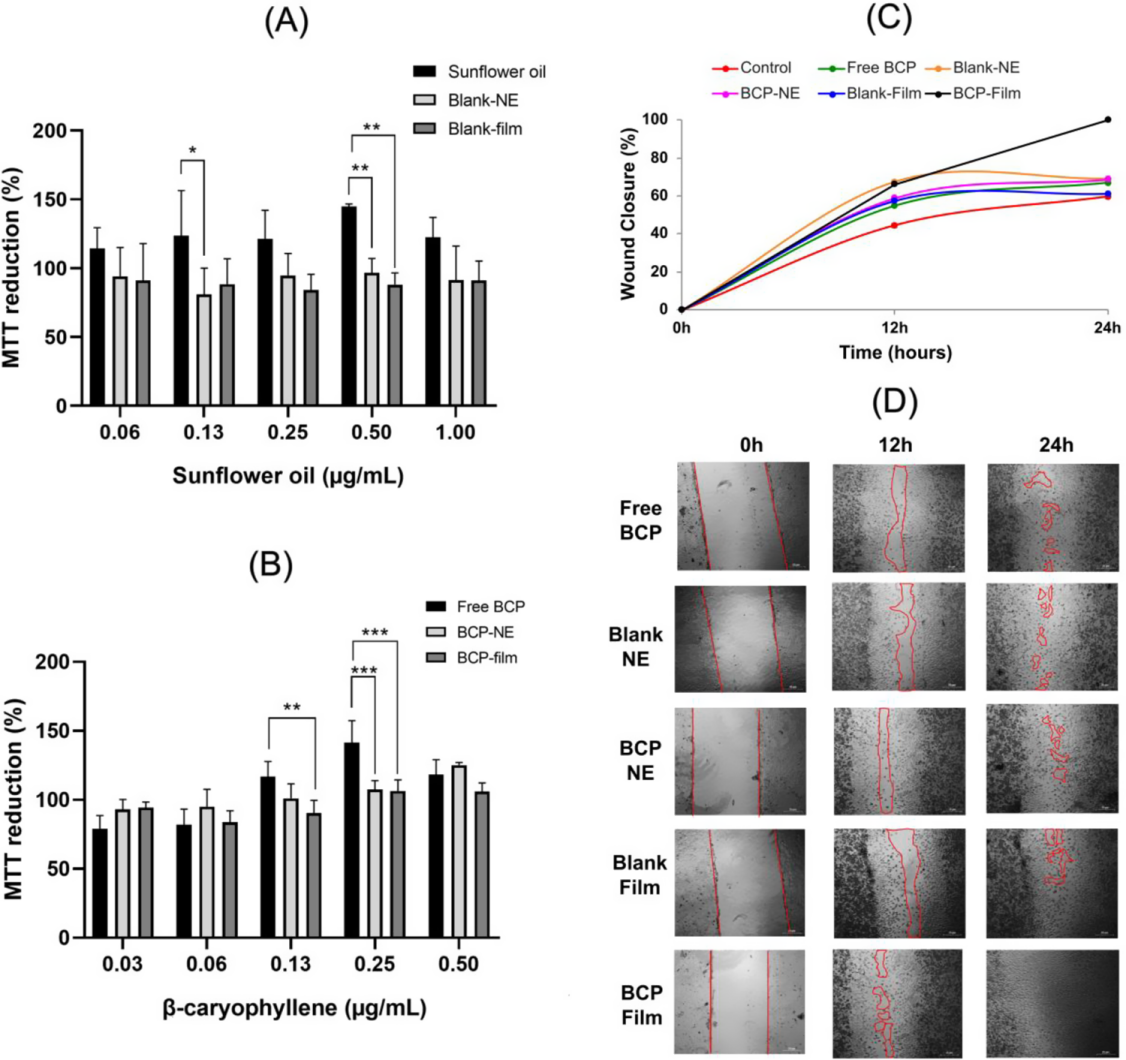
(A) MTT cytocompatibility assay of sunflower oil, Blank-NE, and Blank-Film. (B) MTT cytocompatibility assay of free BCP, BCP-NE, and BCP-Film. Results are expressed as mean ± SD (*n* = 3). Significant differences were determined by ANOVA followed by Tukey’s test (*p < 0.05; **p < 0.01; ***p < 0.001). (C) In vitro wound closure (%) at 0, 12, and 24 h, analyzed by ANOVA with Tukey’s test (*p* < 0.05) or, when needed, by Kruskal–Wallis followed by Dunn’s test. (D) Representative images of L929 fibroblast migration at 0, 12, and 24 h.

The cytocompatibility of formulations containing BCP was also assessed by the MTT assay (Figure 5B). At concentrations of 0.03 and 0.06 μg/mL, no statistically significant differences were observed between the formulations. At 0.13 μg/mL, a significant difference was observed between free BCP and BCP-Film, while at 0.25 μg/mL, free BCP exhibited significantly higher viability compared to BCP-NE and BCP-Film. At the highest concentration tested (0.50 μg/mL), no statistically significant differences were observed between the formulations. Across all tested concentrations, cell viability remained above 78%.

The results of the cell migration assay with L929 fibroblasts are shown in Figure 5C. After 12 h, BCP-Film achieved 73.6 ± 5.3% wound closure, significantly higher than Blank-Film (*p* = 0.005), Free BCP (*p* = 0.008), and BCP-NE (*p* = 0.036), with no significant difference compared to Blank-NE. After 24 h, BCP-Film promoted complete closure (100%), significantly higher than all other treatments (*p* < 0.001). BCP-NE (68.7 ± 1.5%), Blank-NE (66.3 ± 5.0%), Free BCP (63.2 ± 0.3%), and Blank-Film (54.0 ± 2.8%) showed partial closure, with no significant differences among them in several comparisons. Cell migration images (Figure 5D) confirmed that at 12 h, BCP-Film already markedly reduced the wound area, and by 24 h achieved complete closure, whereas the other groups displayed only partial migration.

## 4 Discussion

NEs prepared by spontaneous and catastrophic phase inversion emulsification presented suitable properties for use as drug delivery systems.^13^ Chitosan films containing NE with 1.0% sunflower oil and 0.5% BCP ensured high incorporation efficiency of the sesquiterpene. Physicochemical parameters such as diameter, PdI, and zeta potential indicated that BCP incorporation did not affect the colloidal stability of Blank-NEs, with negative zeta potential values (Blank-NE: −25.5 ± 2.3 mV; BCP-NE: −27.8 ± 0.4 mV) contributing to nanodroplet stabilization.^14^ In addition, the slightly acidic pH (5.4–5.7), together with high encapsulation efficiency and stability over 21 days, reinforces the potential of these systems as stable delivery vehicles for pharmaceutical and cosmetic applications.^15^ In this context, BCP-loaded NEs were used as an intermediate system for subsequent incorporation into chitosan films, ensuring controlled formulation progression from nanosystem to polymeric matrix.

Morphological evaluation by TEM confirmed the formation of NEs, with spherical nanosized droplets observed in both formulations. The presence of smaller droplets within BCP-NE suggests successful incorporation of BCP into the lipid phase, supporting homogeneous dispersion within the final film matrix.

In terms of biological performance, cytocompatibility assays demonstrated that both Blank-NE and BCP-NE are biocompatible with L929 fibroblasts, supporting their suitability for biomedical applications. Blank-NE maintained high cell viability across all conditions, confirming the low toxicity of its components, including sunflower oil, which is widely reported as biocompatible for skin applications.^7^ In contrast, BCP-NE showed a slight reduction in viability at higher concentration, which may be associated with the pharmacological activity of BCP, a sesquiterpene known for anti-inflammatory, antioxidant, and antiproliferative effects.^16^ Importantly, encapsulation within NEs may enhance compound–cell interactions, increasing both therapeutic potential and cytological response modulation.^17^ Nevertheless, cell viability remained above the threshold established by ISO 10993–5, confirming the safety of the system.^18,19^

Complementary hemolysis assays further supported the safety profile of the formulations. While free BCP exhibited marked hemolytic activity, nanoencapsulation significantly reduced this effect, indicating that the NE structure acts as a protective barrier. Blank-NE also demonstrated excellent hemocompatibility, reinforcing the suitability of the excipients for contact with blood or vascularized tissues.

From a functional perspective, antioxidant assays revealed that BCP-NE at 100 μg/mL exhibited higher radical scavenging activity compared to free BCP, suggesting that nanoencapsulation enhances the functional performance of the compound. This effect may be related to improved stability and bioavailability of BCP within the nanocarrier system.^20–22^ Given the central role of oxidative stress in impairing wound healing, these findings are particularly relevant for tissue regeneration strategies.

Following incorporation into polymeric systems, the presence of sunflower oil and BCP increased film mass and thickness, consistent with the incorporation of lipidic phases into the chitosan matrix.^23^ The slightly acidic pH further confirms the suitability of the films for topical application.^24^

Morphologically, SEM analysis revealed marked differences between CH-Film and lipid-containing films. While CH-Film exhibited a compact and homogeneous structure, Blank-Film and BCP-Film presented rough and heterogeneous surfaces with pores and circular domains. These features are likely associated with glycerol plasticization and lipid nanodroplet dispersion within the polymer network. In this context, the increased porosity may favor exudate absorption and interaction with the moist wound environment, which are critical aspects of wound management.^25^

Mechanical evaluation showed no statistically significant differences between Blank-Film and BCP-Film (*p* > 0.05), although BCP-Film exhibited more consistent behavior across replicates. This suggests improved structural uniformity, possibly related to lipid-mediated modulation of polymer chain interactions, which may increase chain mobility and flexibility.^26^

Structural and thermal analyses further confirmed matrix reorganization after lipid incorporation. FTIR, XRD, DSC, and TGA results indicated disruption of the semicrystalline structure of chitosan, leading to amorphization of the system and modification of thermal stability. These changes are consistent with interactions between chitosan, sunflower oil, and BCP, which collectively influence matrix organization and thermal behavior.^27–29^

Biological assays using film systems confirmed their cytocompatibility, with all formulations maintaining acceptable viability levels. Differences observed between free and formulated BCP at intermediate concentrations may be related to variations in compound availability within the different delivery systems, highlighting the role of formulation in modulating biological response while maintaining safety.

Finally, BCP-Film significantly enhanced fibroblast migration, achieving complete wound closure at 24 h. This effect can be attributed to the synergistic action of its components. BCP acts as a selective CB2 receptor agonist, modulating inflammatory responses and promoting fibroblast proliferation and migration.^30^ In parallel, sunflower oil, rich in linoleic acid, contributes to skin barrier restoration and activation of PPAR-α signaling pathways, which are associated with re-epithelialization and angiogenesis.^31^ The NE system enhances BCP stability and bioavailability,while the chitosan matrix provides a biocompatible and structural scaffold that maintains a moist environment and supports cell attachment.^32^ Additionally, the porous morphology of the films may further facilitate exudate management and nutrient exchange, contributing to improved tissue repair conditions.

Although release kinetics were not experimentally evaluated in this study, BCP is expected to become available at the film–wound interface through diffusion and partitioning from the NE dispersed within the chitosan matrix. This behavior may contribute to local compound availability, thereby supporting the observed biological effects. Overall, the combined action of anti-inflammatory modulation, enhanced cell migration, structural support, and favorable microenvironment formation explains the improved wound closure observed with the BCP-Film. Although promising results were obtained in fibroblast migration assays, additional studies addressing inflammatory modulation and angiogenesis are required to further clarify the underlying wound healing mechanisms.

## 5 Conclusions

In this work, a multifunctional and nanostructured wound dressing device was designed and developed, which exhibited satisfactory physicochemical properties, cytocompatibility, and in vitro wound healing activity. BCP-NE (0.5% BCP) showed an average size of 114 nm, low polydispersity (0.136), and high encapsulation efficiency (96%), confirming its ability to incorporate and protect the lipophilic compound. The chitosan film incorporating this nanoemulsion displayed structural uniformity, skin-compatible pH, and 13.3 mg/g BCP content, representing a promising device for wound treatment, achieving complete wound closure in 24 h in vitro and indicating a synergistic effect between chitosan, sunflower oil, and nanoencapsulated BCP. Overall, BCP-Film demonstrates interesting properties for a wound dressing, combining suitable mechanical features for further in vivo studies of therapeutic efficacy in different class of wounds in preclinical and clinical approaches.

## Supplementary Material



## Notes

Photographs documenting the film preparation and development process, gas chromatograms of free BCP, BCP-film, and Blank-film, force–displacement curves for puncture resistance and elongation at break of Blank-film and BCP-film (PDF)

## ABBREVIATIONS

NE: nanoemulsion
BCP: β-caryophyllene
PdI: polydispersity index
SD: standard deviation
EE: encapsulation efficiency
ZP: zeta potential
Blank-NE: empty sunflower oil nanoemulsion
BCP-NE: β-caryophyllene-loaded nanoemulsion
CH-Film: control chitosan film (without nanoemulsions)
Blank-Film: chitosan film containing unloaded sunflower oil-based nanoemulsions
BCP-Film: chitosan film containing β-caryophyllene-loaded nanoemulsions.
