# Supplementary material for: β‑Caryophyllene-Loaded Sunflower Oil Nanoemulsion Incorporated into Chitosan Films for Wound Healing Applications
Source: ACS Omega. 2026 Jun 23;11(26):39019–30. doi: 10.1021/acsomega.6c02735 (PMC13347352; doi:10.1021/acsomega.6c02735)
Supplement: Supplementary file 1 [file ao6c02735_si_001.pdf]

Supporting information

β-caryophyllene-loaded sunflower oil nanoemulsion incorporated into chitosan films for wound healing applications

Renata de Carvalho Feitosa [1], *Beatriz Ketlyn da Cunha Batista* [1], *Thais Mariana Bezerra Tavares* [1], *Thayse Emanuelle Félix Dos Santos* [1], *Addison Ribeiro de Almeida* [1], *Leandro de Santis Ferreira* [1], *Weslley de Souza Paiva* [2], *Hugo Alexandre de Oliveira Rocha* [2], *Anne Emmanuelle Camara Da Silva Melo* [1], *Matheus de Freitas Fernandes-Pedrosa* [2], *Arnóbio Antônio da Silva Júnior* [1*]

[1] Laboratory of Pharmaceutical Technology and Biotechnology (TecBioFar), Graduate Program in Pharmaceutical Sciences, Department of Pharmacy, Federal University of Rio Grande do Norte (UFRN), Natal, Rio Grande do Norte 59012-570, Brazil.

[2] Laboratory of Biotechnology of Natural Polymers (BIOPOL), Department of Biochemistry, Federal University of Rio Grande do Norte (UFRN), Natal, Rio Grande do Norte 59078-970, Brazil.

[*] Corresponding Author.

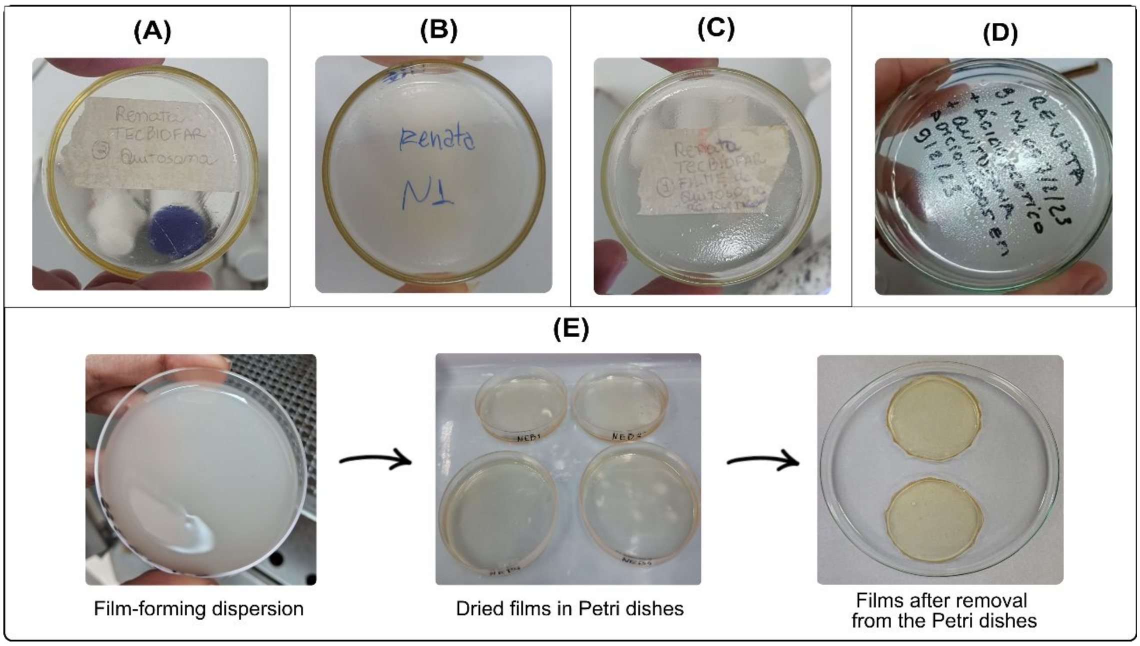

Figure S1. Macroscopic appearance of chitosan-based films: (A) CH-film without nanoemulsion and Blank-films prepared with sunflower oil at (B) 1.0%, (C) 2.5%, and (D) 5.0%. (E) Representative images of the film preparation process, including the film-forming dispersion, dried films in Petri dishes, and films after removal.

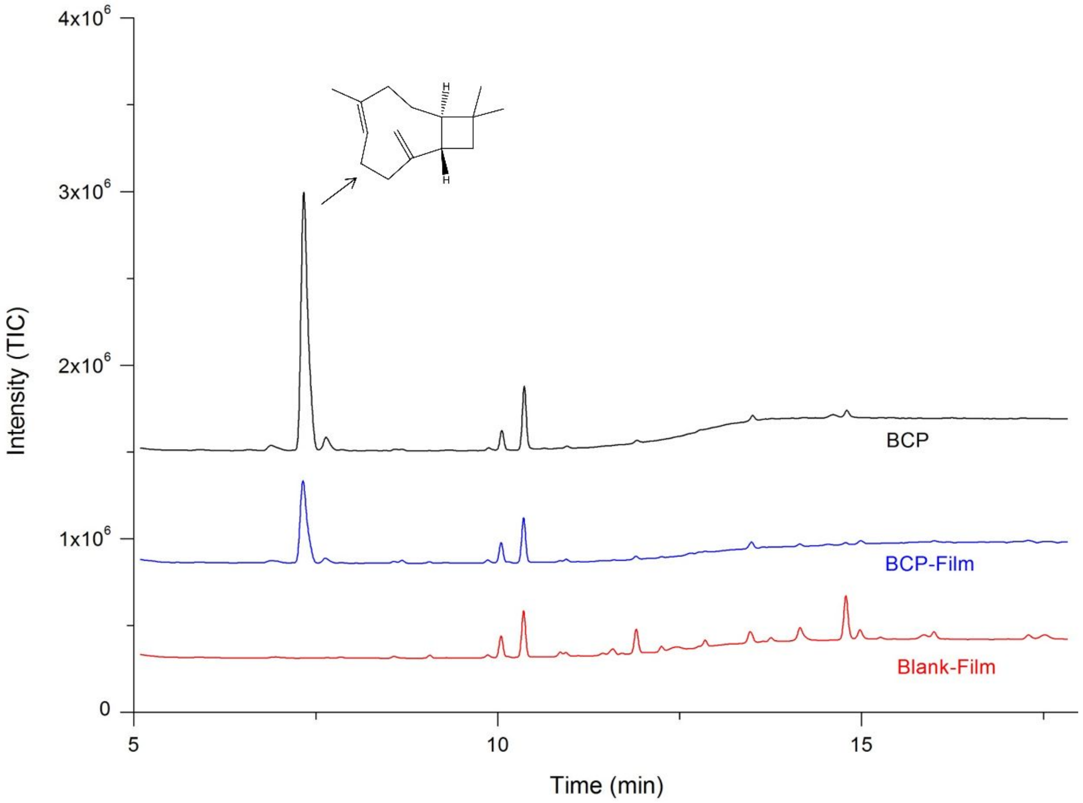

Figure S2. Chromatogram of free BCP, BCP-film and Blank-film samples obtained by gas chromatography.

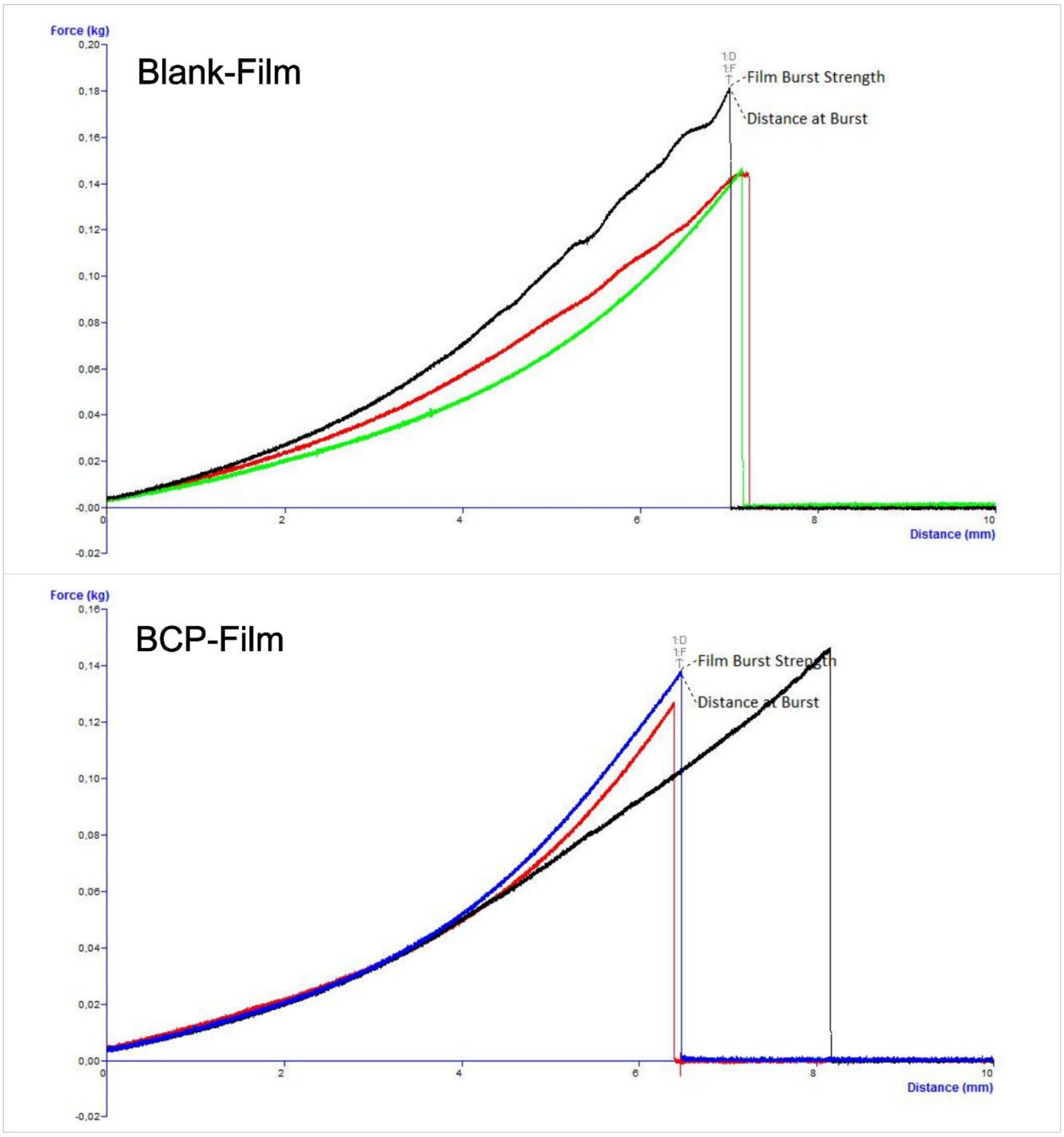

Figure S3. Force (kg) versus displacement (mm) curves recorded until rupture for Blank-Film and BCP-Film in the puncture resistance (Rp) and elongation at break (EB) tests.
